# Increased Demand for Therapeutic Drugs in Pediatric Ulcerative Colitis Patients With Extraintestinal Manifestations

**DOI:** 10.3389/fped.2022.853019

**Published:** 2022-05-23

**Authors:** Yiyoung Kwon, Eun Sil Kim, Yon Ho Choe, Mi Jin Kim

**Affiliations:** Department of Pediatrics, Samsung Medical Center, School of Medicine, Sungkyunkwan University, Seoul, South Korea

**Keywords:** ulcerative colitis, children, extraintestinal manifestation, relapse, drugs demand

## Abstract

**Background:**

Ulcerative colitis (UC) is a systemic inflammatory disease with a gut predominance, which may involve other organs. The presence of extraintestinal manifestation (EIM) is an important symptom for clinicians as it alters the treatment decisions. In this study, we aimed to evaluate the initial clinical presentation and disease severity of pediatric UC patients with EIMs.

**Methods:**

One hundred forty-two patients under the age of 18 years who were diagnosed with UC from January 2003 to November 2021 were included in this study. Forty-seven patients with confirmed EIMs and 95 patients without EIMs were divided into two groups and their differences were analyzed.

**Results:**

The most common EIM was peripheral arthritis. The disease extent at the time of diagnosis shows a higher rate of pancolitis in the EIM-positive group (65.9%) than that of the EIM-negative group (33.7%) (*p* < 0.001). More than 90% of EIM-positive patients had moderate to severe disease activity on the Mayo endoscopic subscore. In the EIM-positive group, the cumulative use of systemic steroids, immunosuppressants, and biological agents from diagnosis to 1 year follow-up were significantly higher than those of the EIM-negative group (*p* = 0.009, 0.001, and < 0.001, respectively). About 80% of patients in the EIM-negative group reached remission, but only about 50% of the EIM-positive patients reached remission (*p* = 0.005). The relapse occurred more frequently in the EIM-positive group than in the EIM-negative group with statistical significance (*p* < 0.001).

**Conclusion:**

Pediatric UC with EIMs had higher disease severity and often manifested upper gastrointestinal tract involvement. Despite EIMs treatment, the occurrence of new EIMs was observed repeatedly. Cumulative drug demand (steroids, immunosuppressants, and biological agents) for the treatment increased steadily over time, and frequent relapses occurred despite the combinatory use of therapeutic drugs.

## Introduction

Ulcerative colitis (UC) is a chronic inflammatory bowel disease (IBD) of which etiology remains unknown (1), but a potential cause includes immune system dysfunction (2). Since the immune system overacts in the body, features of autoimmunity develop in patients with IBD. Therefore, UC can be classified as a systemic inflammatory disease with a gut predominance, which may involve other organs. The clinical presentation of invasion to other organs in IBD patients is called extraintestinal manifestation (EIM), which is expressed in 25–40% of patients. The common expression regions include joints, skin, eyes, kidneys, and liver (3). In rare cases, the biliary tract, pancreas, and ear can also be involved, and hematologic disorders such as anemia and immunothrombocytopenia (ITP) can occur. As the prevalence of EIMs was found to be higher in Crohn’s disease (CD) than in UC, (4) many studies on EIM in IBD have focused more on CD than UC. Immunological differences of CD and UC may be considered as the cause of differences in these EIMs rates. Tolerizing regulatory T cells (Tregs) and pro-inflammatory Th17 cells have been evaluated as newly emerging immune systems associated with IBD in recent studies, but traditionally Crohn’s disease is associated with a Th1 cytokine profile, whereas Th2 cytokines are modulators of ulcerative colitis (5).

In the majority of studies, EIM is found to be associated with disease severity and needs of therapy escalation (6, 7). In addition, patients with EIMs are known to have worse long-term disease outcomes (8). Therefore, the presence of EIMs is an important factor for clinicians to consider as it alters the treatment decisions and may require consultation with other clinical departments. It is necessary to evaluate the clinical presentation and observe clinical courses long-term in pediatric UC patients with EIMs.

In this study, we aimed to evaluate the initial clinical presentation and disease severity of pediatric UC patients with EIMs. Furthermore, we aimed to compare the cumulative relapses of patients with and without EIMs as a long-term disease outcome. Finally, if the relapse rate was different in the two groups, we aimed to evaluate the drug demand (steroids, immunosuppressants, and biological agents) for the treatment.

## Materials and Methods

### Patients

**FIGURE 1:**
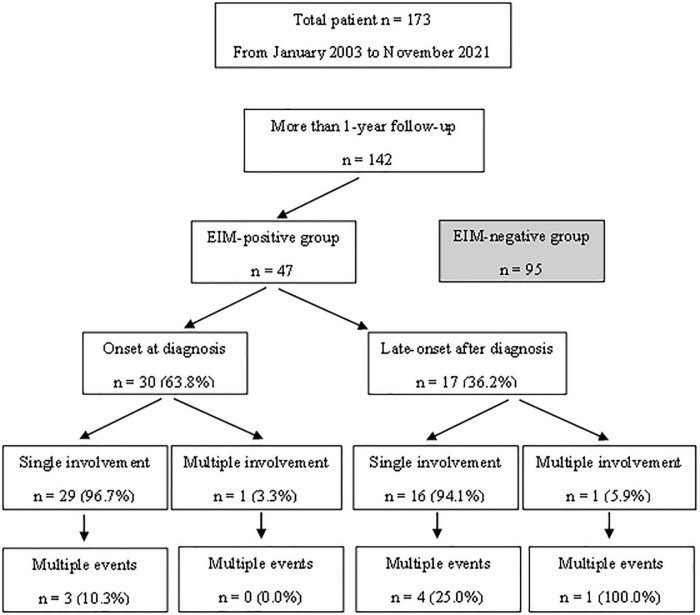
A flow chart diagram showing the subject selection processes and the characteristics of patients with extraintestinal manifestations.

Patients under the age of 18 years who were diagnosed with UC and monitored from January 2003 to November 2021 were included in this study. Although the initial number of patients was 173, patients who were observed for less than a year were eliminated, resulting in 142 patients selected as the study group (Figure 1). All patients were children or adolescents under the age of 18 years at the time of diagnosis, but some patients became adults during the follow-up period. UC and atypical UC was diagnosed following the guidelines of the European Society for Pediatric Gastroenterology, Hepatology and Nutrition (ESPGHAN) (the Porto criteria) (9). This study was approved by the Institutional Review Board of Samsung Medical Center (IRB File No. SMC 2021-12-062).

### Data Collection and Study Design

Data were retrospectively collected. Data at diagnosis include age, laboratory results, pediatric ulcerative colitis activity index (PUCAI), and the extent and severity of the disease. All data from UC and atypical UC patients were collected, but patients with IBD unclassified were excluded. Based on the colonoscopy findings, disease extent was classified into E1 (proctitis), E2 (left colitis), E3 (right colitis), and E4 (pancolitis) according to the Paris classification (10). Upper gastrointestinal (UGI) tract involvement in atypical UC patients was investigated. The disease severity was classified from 0 to 3, using the Mayo endoscopic subscore (MES) (11). Since the fecal calprotectin test has been available in Korea since 2017, it has not been included as a comparative test.

Laboratory results, PUCAI, and the extent and severity of disease were evaluated again 1 year after the diagnosis. In addition, usage of medical drugs including systemic steroids, immunosuppressants (azathioprine, methotrexate, and cyclosporine), and biologic agents (infliximab, adalimumab, and golimumab), and the treatment duration (data collection of start date and end date) were investigated and recorded. Treatment was basically performed as a step-up treatment. In all patients, mesalazine treatment was attempted from the beginning, and the majority of patients started the drug at a dose of 40–60 mg/kg/day. Most of the patients with pancolitis findings and mayo score of 3 used systemic steroids. When used, 1 mg/kg/day was started and tapering was performed at intervals of 10–14 days. Immunosuppressants were added to patients who did not achieve remission after that, and azathioprine at a dose of 0.5–1.0 mg/kg was used. The use of infliximab for pediatric UC has been approved since 2012 in Korea. Prior to 2012, repeated systemic steroids were used in cases of relapse. However, after 2012, infliximab was used when there was no response or dependence on steroids.

The diagnosis of EIM was referred to the hospital records and outpatient records. Records from other departments such as orthopedics, ophthalmology, and rheumatology were also evaluated. The case involving the UGI tract was also counted as a type of atypical UC. All EIMs and UGI involvement occurring after the time of UC diagnosis were recorded. 47 patients with confirmed EIMs or UGI involvement and 95 patients without EIMs were divided into two groups and the differences were analyzed.

### Evaluation of Clinical Outcome

Colonoscopy was performed at the time of diagnosis and 1 year after initiation of treatment. After that, colonoscopy or sigmoidoscopy was performed when relapse was suspected or with an interval of 1–2 years in patients without relapse during the follow-up period. Remission was defined as endoscopic mucosal healing, which indicates no lesion observation and the MES is 0 or 1.

To compare the clinical course between groups, the cumulative relapses that occurred during the follow-up period was investigated. All of the cases where multiple relapses were observed in one patient during the follow-up period were counted. Clinical relapse was defined as a PUCAI score of >10 with modification of treatment. Treatment target was also defined as clinical remission with PUCAI score of <10. Situations in which symptoms temporarily worsened due to infections such as gastroenteritis were excluded *via* stool exams.

### Statistical Analysis

**FIGURE 2:**
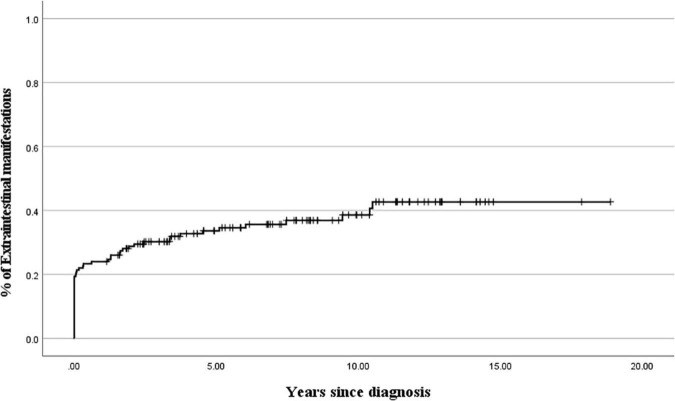
Cumulative probability of extraintestinal manifestations development during 10 years after the diagnosis of ulcerative colitis.

**FIGURE 3:**
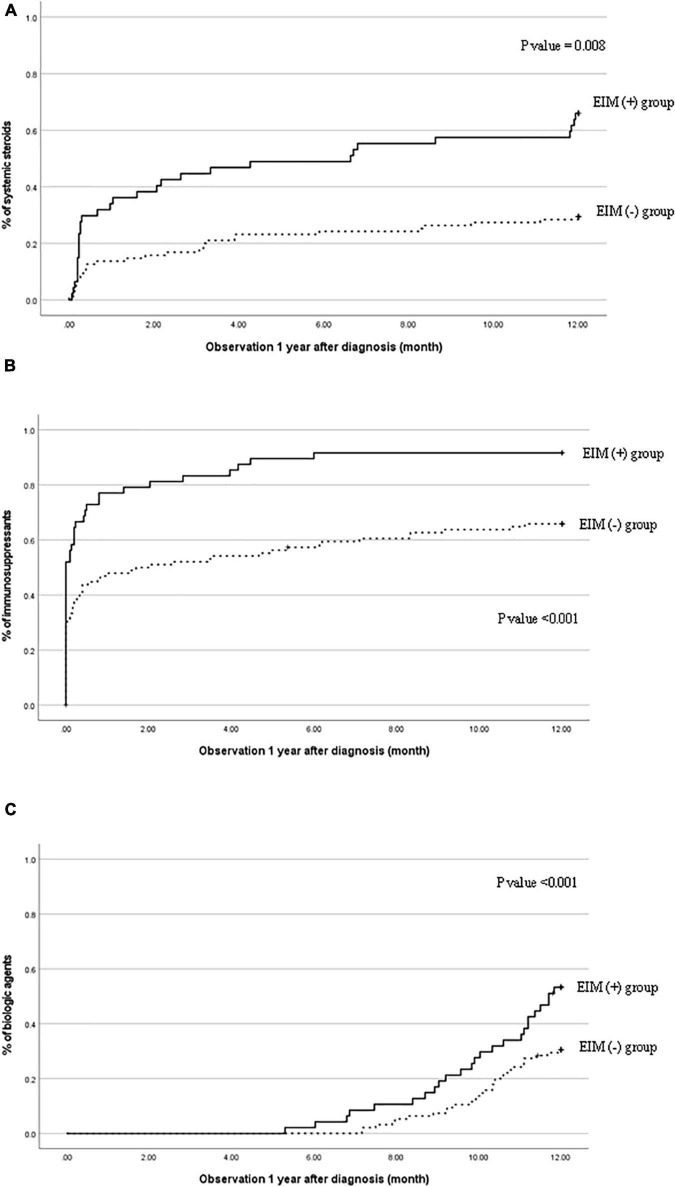
Cumulative probability of **(A)** systemic steroids, **(B)** immunosuppressants, and **(C)** biological agents in patients with EIM (+) and patients with EIM (−). EIM, extraintestinal manifestation.

For descriptive statistics, continuous variables were expressed as average (standard deviation) and categorical variables as absolute numbers with percentages. Comparisons between groups were made by the Student’s t-test for continuous variables and by the χ^2^ test for categorical variables (Tables 2, 3). Kaplan-Meier survival plots were used for descriptive time-dependent data (Figures 2, 3, 5).

**TABLE 1:** Clinical characteristics and the onset time of extraintestinal symptoms of patients in the EIM (+) group.

Variables	Patients no.	Age	Sex (M/F)	Onset time (year) from diagnosis
UGI tract involvement	34 (72.3)	15.9 (14.1–17.2)	19 (55.9)/15 (46.9)	0.00 (0.00–0.08)
Peripheral arthritis	8 (17.0)	14.4 (12.4–15.7)	2 (25.0)/6 (75.0)	1.50 (0.27–2.69) One—before diagnosis
Sacroiliitis	4 (8.5)	10.4 (9.4–12.8)	1 (25.0)/3 (75.0)	0.16 (0–0.71)
Spondyloarthropathy	2 (4.3)	15.3 (14.7–15.9)	1 (50.0)/1 (50.0)	7.75 (6.43–9.08)
Pyoderma gangrenosum	2 (4.3)	16.7 (15.7–17.6)	1 (50.0)/1 (50.0)	0.05 (0.02–0.07)
Immune thrombocytopenia	2 (4.3)	15.4 (15.2–15.7)	2 (100)/0 (0.0)	0.03 (0.01–0.04)
Primary sclerosing cholangitis	1 (2.1)	16.4	M	At diagnosis
Ear involvement	1 (2.1)	14.1	F	1.19
Thyroid involvement	1 (2.1)	9.4	F	3.73
Pancreatitis	1 (2.1)	16.1	M	At diagnosis
Myositis	1 (2.1)	14.7	F	Before diagnosis
Uveitis	1 (2.1)	11.4	M	9.44
Deep vein thrombosis	1 (2.1)	17.5	M	At diagnosis

*EIM, Extraintestinal manifestation; UGI, Upper gastrointestinal tract involvement.*

*Values are presented in “n (percentage)” or “median (interquartile range).”*

**TABLE 2:** Demographic and clinical features at the time of diagnosis of the two groups; patients in EIM (+) group have extraintestinal manifestations with intestinal symptoms and patients in EIM (−) group only express intestinal symptoms.

		EIM (+) group (*N* = 47)	EIM (−) group (*N* = 95)	*P*-value
Age at diagnosis, years		14.96 ± 2.68	14.13 ± 3.26	0.110^c^
Total duration of follow up, years		7.74 ± 2.94	7.78 ± 4.10	0.953^c^
PUCAI^a^ at diagnosis		45.64 ± 15.76	38.95 ± 16.73	**0.022** ^c^
Hematocrit at diagnosis, g/dl		33.36 ± 7.84	37.41 ± 6.12	**0.001** ^c^
Albumin at diagnosis, g/dl		4.08 ± 0.53	4.32 ± 0.49	**0.012** ^c^
ESR at diagnosis, mm/h		36.87 ± 29.25	19.69 ± 21.49	**<0.001** ^c^
CRP at diagnosis, mg/dl		1.16 ± 2.15	0.38 ± 1.02	**0.004** ^c^
Disease extent of Paris classification at diagnosis				**<0.001** ^d^
E1	Proctitis	7 (14.9)	33 (34.7)	
E2	Left colitis	2 (4.3)	18 (18.9)	
E3	Extensive colitis	7 (14.9)	12 (12.6)	
E4	Pancolitis	31 (65.9)	32 (33.7)	
Mayo endoscopic subscore at diagnosis^b^				**0.001** ^d^
1	Mild	2 (4.3)	20 (21.1)	
2	Moderate	30 (63.8)	62 (65.3)	
3	Severe	15 (31.9)	13 (13.7)	

*EIM, Extraintestinal manifestation; N, number of patients.*

*Values are represented in “n (percentage)” or “average ± standard deviation.”*

*^a^Pediatric Ulcerative Colitis Activity Index (PUCAI) is a 6-item disease activity index intended for use in pediatric UC clinical trials with a score ranging from 0 – 85.*

*^b^Mayo endoscopy subscores were as follows: 0, normal or inactive disease; 1, mild disease; 2, moderate disease; and 3, severe disease.*

*^c^Student t-test.*

*^d^χ^2^ test. Bold values mean statistically significant values with p-value < 0.05.*

**TABLE 3:** Demographic and clinical features of the two groups 1 year after the diagnosis; patients in EIM (+) group have extraintestinal manifestations with intestinal symptoms and patients in EIM (−) group only express intestinal symptoms.

		EIM (+) group (*N* = 47)	EIM (−) group (*N* = 95)	*P*-value
PUCAI^a^		13.80 ± 16.94	13.05 ± 12.95	0.791^c^
Hematocrit, g/dl		38.22 ± 5.17	40.07 ± 3.84	**0.019^c^**
Albumin, g/dl		5.14 ± 0.42	4.46 ± 0.37	0.223^c^
ESR, mm/h		30.72 ± 28.73	18.25 ± 16.07	**0.001^c^**
CRP, mg/dl		0.52 ± 0.95	0.22 ± 0.45	**0.011^c^**
**Disease extent of Paris classification**				
None		7 (14.9)	32 (33.7)	**0.001^d^**
E1	Proctitis	11 (23.4)	28 (29.5)	
E2	Left colitis	6 (12.8)	11 (11.6)	
E3	Extensive colitis	11 (23.4)	15 (15.8)	
E4	Pancolitis	12 (25.5)	9 (9.5)	
Mayo endoscopic subscore^b^				**0.005^d^**
0	Normal or inactive	10 (21.3)	33 (34.7)	
1	Mild	15 (31.9)	41 (43.2)	
2	Moderate	19 (40.4)	20 (21.1)	
3	Severe	3 (6.4)	1 (1.1)	
Systemic corticosteroid		25 (53.2)	29 (30.5)	**0.009^d^**
Immunosuppressants		43 (91.5)	62 (65.3)	**0.001^d^**
Biologic agents		31 (66.0)	28 (29.5)	**<0.001^d^**
**EIM improvement**				
Yes		44 (93.6)		
No		3 (6.4)		

*EIM, Extraintestinal manifestation; N, number of patients.*

*Values are represented in “n (percentage)” or “average ± standard deviation.”*

*^a^Pediatric Ulcerative Colitis Activity Index (PUCAI) is a 6-item disease activity index intended for use in pediatric UC clinical trials with a score ranging from 0 – 85.*

*^b^Mayo endoscopy subscores were as follows: 0, normal or inactive disease; 1, mild disease; 2, moderate disease; and 3, severe disease.*

*^c^Student t-test.*

*^d^χ^2^ test. Bold values mean statistically significant values with p-value < 0.05.*

**FIGURE 4:**
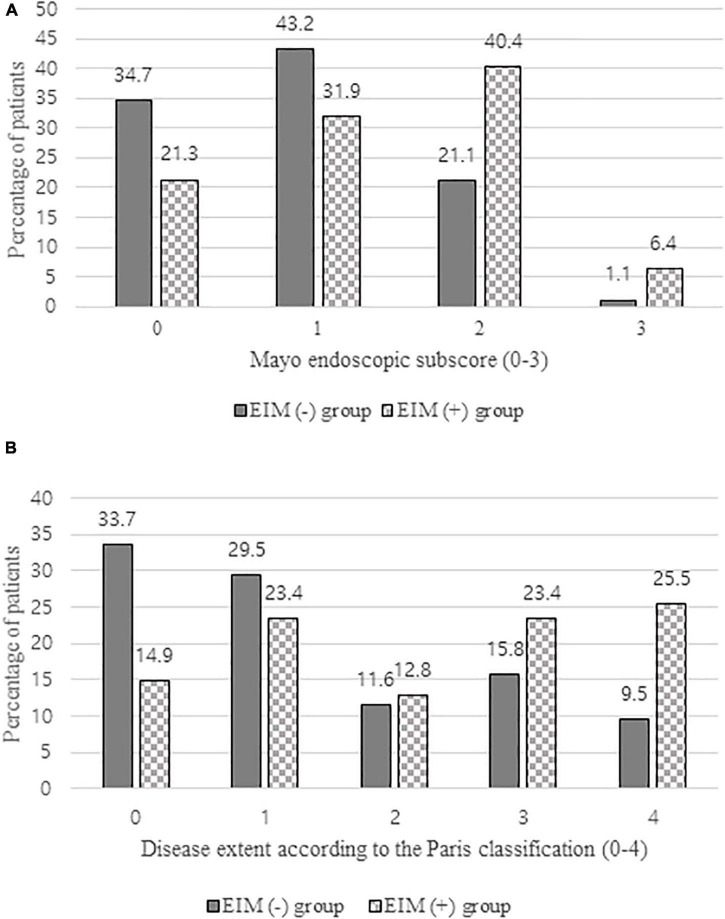
**(A)** Comparison of mayo endoscopic subscore of EIM (+) group and EIM (−) group evaluated by colonoscopy 1 year after the diagnosis. **(B)** Comparison of disease extent of EIM (+) group and EIM (−) group according to the Paris classification evaluated by colonoscopy 1 year after the diagnosis. EIM, extraintestinal manifestation.

**FIGURE 5:**
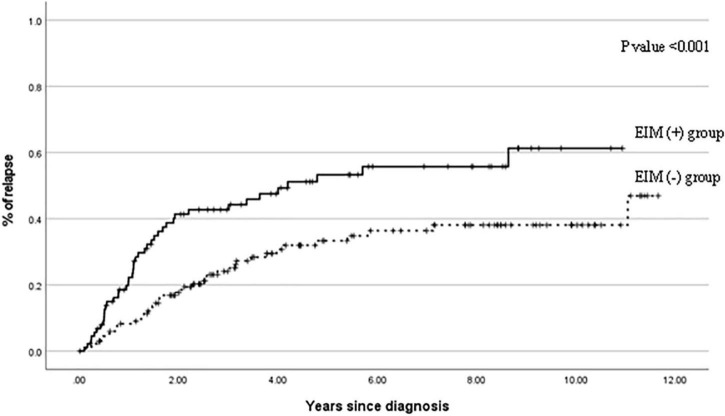
Cumulative probability of relapse events in patients with EIM (+) and patients with EIM (−) over 10 years after the diagnosis. EIM, extraintestinal manifestation.

All of the above statistical analyses were conducted using SPSS version 27 (IBM Corporation, Armonk, NY, United States). A *p*-value less than 0.05 was considered statistically significant.

## Results

### Baseline Characteristics

Table 1 shows the clinical characteristics of patients with EIMs and the onset time of EIMs. All EIM is described in detail, rather than broadly categorized as joint or skin. The highest occurrence rate of atypical features was UGI tract involvement, which is frequently observed in CD, not UC. The endoscopic findings of UGI included friable mucosa, multiple mucosal erosions, mucosal edema, and superficial or deep ulcers in the esophagus, stomach, and duodenum. As pathological findings, only inflammation and ulceration in which *Helicobacter pylori* was excluded were included. The highest occurrence rate of EIMs was peripheral arthritis, which involves knee joints, ankle, arm, or hand. In cases where the joint symptoms were manifested as both sacroiliitis and spondyloarthropathy, the proportion of females was higher than that of men. Pyoderma gangrenosum and ITP were observed in two patients. Biliary tract, ear, thyroid, eye involvement, pancreatitis, myositis, and deep vein thrombosis were observed in each patient. These EIMs were present in some patients at the time of diagnosis, but in some patients, they were not present at the time of diagnosis, but they were newly developed while undergoing relapse during treatment. In addition, there were cases where the EIMs present at the time of diagnosis improved after treatment and then recurred (Figure 1). Cumulative EIMs during the total follow-up period for patients in this study are shown in Figure 2.

Table 2 compares the clinical characteristics at diagnosis of the EIM-positive group and the EIM-negative group. While there were no statistically significant differences in age and follow-up period between the two groups, characteristics inferring the severity of the disease showed differences. The clinical symptom score, PUCAI, was significantly higher in the EIM-positive group than in the negative group (45.64 vs. 38.95, *p* = 0.022). Hematocrit (33.36 vs. 37.41, *p* = 0.001) and albumin (4.08 vs. 4.32, *p* = 0.012) levels of the EIM-positive group were also significantly lower in laboratory results, suggesting that bleeding and diarrhea were more severe. Conversely, ESR (36.87 vs. 19.69, *p* < 0.001) and CRP (1.16 vs. 0.38, *p* = 0.004), which indicate the level of inflammation, were significantly higher in the EIM-positive group. The disease extent at the time of diagnosis evaluated by colonoscopy shows a higher rate of pancolitis in the EIM-positive group (65.9%) than in the negative group (33.7%) with a statistical significance (*p* = < 0.001). 95.7% of EIM-positive patients had moderate to severe disease on the MES, whereas 79.0% of EIM-negative patients were in moderate to severe disease status (*p* = 0.001).

### Increased Demand for Therapeutic Drugs

Figure 3 shows the necessities of systemic steroids, immunosuppressants, and biological agents 1 year after the diagnosis were evaluated cumulatively in the EIM-positive and EIM-negative groups. In the EIM-positive group, the use of all three drugs was significantly higher than that of the EIM-negative group (*p* = 0.008, <0.001, and <0.001, respectively). Since infliximab was approved for use in pediatric UC in 2012, infliximab has substituted for therapeutic drugs instead of systemic steroids when there were relapses. Therefore, Looking at the graphs in Figure 3, the slope of the graph of systemic steroid (A) in the second half decreases, while the slope of the graph of biological agents (C) increases in the latter half.

The clinical characteristics evaluation after 1 year of diagnosis also shows the rate of drug use at 1 year (Table 3). Within a year of diagnosis, 53.2% of patients in the EIM-positive group had used systemic steroids, 91.5% of patients started using immunosuppressants, and 66% of patients started using biological agents for treatment.

### Comparison of Clinical Outcomes

As illustrated by Table 3, patients in both groups exhibited lower PUCAIs after 1 year after the diagnosis and the initiation of treatment with one or more therapeutic drugs, which were not significantly different between the two groups. In laboratory results, no statistically significant difference was observed in albumin level, whereas the hematocrit level was significantly lower and ESR and CRP were significantly higher in the EIM-positive group (*p* = 0.019, 0.001, and 0.011, respectively). 93.6% of patients with EIM had improved their symptoms of EIM, but three of them still needed some medications for EIMs even after the treatment. These three patients will be discussed later in the discussion part. Looking at the results of colonoscopy, more than 60% of patients in the EIM-negative group who initially showed a mild disease status either disappeared (33.7%) or proctitis (29.5%), but in the EIM-positive group, about 50% of patients still had extensive colitis (23.4%) or pancolitis (25.5%). In terms of the severity, about 80% of patients in the EIM-negative group reached remission with MES grade 0 (normal or inactive, 34.7%) or grade 1 (mild, 43.2%), but only about 50% of patients reached remission in the EIM-positive group. There were statistically significant differences in disease extent and severity between the two groups (*p* = 0.001 and 0.005, respectively) (Figure 4).

Figure 5 shows the cumulative relapse during the follow-up period. The relapse occurs more frequently in the EIM-positive group than in the EIM-negative group with a statistical significance (*p* ≤ 0.001).

Only one patient who was diagnosed with EIM of deep vein thrombosis underwent a colectomy during the follow-up period. The patients underwent total proctocolectomy 3 years after the diagnosis due to uncontrolled hematochezia, despite the effort to control the hematochezia using mesalazine, azathioprine, methylprednisolone, antibiotics, and infliximab.

### Differences According to When Extraintestinal Manifestation Occurs

Since the analyzes so far have analyzed both patients who had EIM from the time of diagnosis and patients who developed EIM later, we also tried to evaluate whether the demographic and clinical features at the time of diagnosis differ depending on the time of EIM. Table 4 shows the comparison of 47 patients who had EIM divided into 30 patients who had EIM from the time of diagnosis and 17 patients who developed EIM later. The factor that showed statistically significant difference was only the mayo endoscopic subscore, but the higher values of the PUCAI (48.33 vs. 40.88), ESR (39.60 vs. 32.06), and CRP (1.41 vs. 0.71) was observed in the patient group who had EIM from the time of diagnosis. The hematocrit (32.75 vs. 34.44) and albumin (3.97 vs. 4.27) values were also lower in the patient group who had EIM from the time of diagnosis. These results suggest that patients with EIM at the time of diagnosis had worse initial clinical features than patients who developed EIM later. In the mayo endoscopic subscore, which showed a statistically significant difference, the proportion of patients with severe status was 3.7 times higher in patients with EIM from the time of diagnosis (43.3 vs. 11.8%).

**TABLE 4:** Demographic and clinical features at the time of diagnosis of the two groups; patients with EIM at the time of diagnosis and patients who had developed EIM later.

		EIM at diagnosis (*N* = 30)	EIM later (*N* = 17)	*P*-value
Age at diagnosis, years		14.93 ± 2.93	15.02 ± 2.27	0.919^c^
Total duration of follow up, years		6.86 ± 2.98	6.95 ± 3.33	0.925^c^
PUCAI^a^ at diagnosis		48.33 ± 15.28	40.88 ± 15.93	0.121^c^
Hematocrit at diagnosis, g/dl		32.75 ± 8.37	34.44 ± 6.92	0.483^c^
Albumin at diagnosis, g/dl		3.97 ± 0.51	4.27 ± 0.54	0.060^c^
ESR at diagnosis, mm/h		39.60 ± 32.25	32.06 ± 23.15	0.402^c^
CRP at diagnosis, mg/dl		1.41 ± 2.50	0.71 ± 1.30	0.291^c^
Disease extent of Paris classification at diagnosis				<0.511^d^
E1	Proctitis	3 (10.0)	4 (23.5)	
E2	Left colitis	2 (6.7)	0	
E3	Extensive colitis	5 (16.7)	2 (11.8)	
E4	Pancolitis	20 (66.7)	11 (64.7)	
Mayo endoscopic subscore at diagnosis^b^				**0.036** ^d^
1	Mild	1 (3.3)	1 (5.9)	
2	Moderate	16 (53.3)	14 (82.4)	
3	Severe	13 (43.3)	2 (11.8)	

*EIM, Extraintestinal manifestation; N, number of patients.*

*Values are represented in “n (percentage)” or “average ± standard deviation.”*

*^a^Pediatric Ulcerative Colitis Activity Index (PUCAI) is a 6-item disease activity index intended for use in pediatric UC clinical trials with a score ranging from 0 to 85.*

*^b^Mayo endoscopy subscores were as follows: 0, normal or inactive disease; 1, mild disease; 2, moderate disease; and 3, severe disease.*

*^c^Student t-test.*

*^d^χ^2^ test. Bold values mean statistically significant values with p-value < 0.05.*

## Discussion

We retrospectively studied the clinical characteristics and the demand for therapeutic drugs in pediatric UC with EIMs. Knowing the features of EIMs initially and the disease progression in patients with EIMs is important because clinicians’ treatment decisions may differ, in turn, predict long-term outcomes.

Our major findings were as follows. First, the intestinal disease severity at the time of diagnosis was more severe in UC patients with EIMs. Second, the UGI tract is often invaded in the case of pediatric ulcerative colitis patients. Third, even patients who did not have EIMs at the time of diagnosis may develop EIMs although they were taking maintenance medications with experiencing relapse. Fourth, UC patients with EIMs significantly increased the drug demand for treatment compared to UC patients without EIMs. Fifth, even with many medications, UC patients with EIM do not achieve remission well and experience frequent relapses even after reaching remission. Taken together, our findings suggest that use of biological agents as an induction treatment should be considered in UC patients with EIM, and clinicians should check carefully whether there are newly developed EIMs that patients are not aware of, even during maintenance treatment. Moreover, in addition to the colonoscopy, esophagogastroduodenoscopy (EGD) should be performed at the time of diagnosis. Finally, a variety of biological agents have been released recently, thus, understanding the mechanisms and the effects of each biological agent helps select a treatment strategy for EIMs.

UGI tract involvement is more common in CD than in UC, and it is also known that it occurs more frequently in children than adults in CD (12). Recently, studies of CD report that South Asians have more UGI tract involvement than Westerners (13), which were explained by the genetic differences according to race and the influence of food culture (14, 15). As an extension of these studies, emerging evidence suggested that UC also presents complications in the UGI tract (16, 17). Therefore, UGI tract involvement was previously considered to be a CD-specific finding, but it can no longer be used to distinguish CD from UC as it may be seen in both entities. Both ESPGHAN and European Crohn’s and colitis organization recommend EGD especially for pediatric patients (10, 11). The ESPGHAN especially emphasizes performing EGD in all children irrespective of presence or absence of UGI symptoms and performing multiple biopsies even when the macroscopic features are normal (9). Invasion of the UGI tract is a phenotype of atypical UC, but the lesion pattern is different from that of CD. The lesions are erosions or small ulcers, but are neither serpiginous nor linear. A Biopsy also reveals diffuse or focal gastritis without granuloma (18). In a recently published review article, when reviewing UC patients who had lesions in the UGI tract, it was confirmed that patients with focally enhanced gastritis accounted for 21–30% of all UCs, and it was reported that lymphohistiocytes were mainly observed with focal pit injury when histologically evaluated (14, 19, 20). This study also confirmed the presence of UGI tract involvement in children with ulcerative colitis. This fact highlights the necessity to evaluate the patient through regular questioning for the upper gastrointestinal symptoms even during follow up and performing EGD.

Although EIMs are most often detected at the time of diagnosis, they can continue to develop new symptoms throughout the treatment. Recent studies have revealed that children diagnosed with EIM are more likely to develop persistent EIMs during the treatment than adults (21, 22). Patients in this study also developed new EIMs of different patterns together with worsened intestinal symptoms even after the treatment. Rarely, the symptoms of EIM developed before UC, making the diagnosis of UC difficult. Among our patients, two patients showed EIMs symptoms before intestinal symptoms. One was diagnosed with juvenile rheumatoid arthritis (JRA) for peripheral arthritis symptoms, and was being treated with methotrexate. The other patient developed fever and myositis, and was hospitalized in the infection department first. The patient who was treated with JRA developed intestinal symptoms after a year and was diagnosed with UC. Among the patients with intestinal manifestation in JRA, those with clearly IBD often have a family history of IBD and do not improve with typical disease-modifying antirheumatic drugs, but improve with biological agents used for IBD (23). The patient who had been treated for myositis developed intestinal symptoms 2 months later, and was diagnosed with UC. These are rare cases but if the criteria for other autoimmune diseases are not clearly satisfied in patients who develop EIMs first, it is necessary to suspect IBD and consider performing stool calprotectin, occult blood test, or colonoscopy.

Treating patients with EIMs is a major challenge for clinicians. Many studies mentioned the importance of systemic steroids and anti-TNF alpha for the treatment of EIMs. However, steroids cannot be continued for long-term use in children because they cause osteopenia and growth delay. In addition, since measurement of anti-TNF alpha drug and antibody concentrations have recently become possible, and other biological agents have been developed, various treatment options should be considered. Although the newly developed biological agents such as ustekinumab or tofacitinib have not yet been approved in children, adult UC studies have confirmed that they can be used for the treatment of EIM (24–26). Evaluating other drugs, non-steroidal anti-inflammatory drug (NSAID) may be used as in patients with JRA because there is insufficient evidence to warrant NSAID avoidance among those IBD patients who really need them for joint symptoms (27). Sulfasalazine has been shown in several studies to be effective in AS and it may be effective on peripheral joint involvement (28). Although methotrexate is not a commonly used immunosuppressant in UC, it may be effective in AS. In addition, immunosuppressants such as 6-mercaptopurine, cyclosporine, and tacrolimus may be used for EIM treatment.

We had three patients who had lifelong sequelae after EIMs and had difficulties in treating EIMs, and they suffered from the following causes. One patient had thyroid involvement and continued to take levothyroxine due to persistent hypothyroidism as sequelae. If the function of the thyroid has already been decreased after the occurrence of EIMs, it must remain as lifelong sequelae because the function cannot be restored even after the usage of steroids or biological agents. Another patient had severe pyoderma gangrenosum on the face and body, and experienced repeated wax and wane after trying infliximab, adalimumab, steroids, and immunosuppressants such as cyclosporine and azathioprine. The patient finally became an adult and the symptoms ameliorated after changing the biological agent to ustekinumab. Ustekinumab is a drug originally used for psoriasis and is evaluated to have a good effect in treating EIMs on the skin (29–31). The last cause was peripheral arthritis in various parts of the body. The patient also tried steroids and various immunosuppressants, but the symptom repeated continuously. We also tried infliximab, the only biological agent available, but it didn’t improve (In Korea, only infliximab can be used in UC patients under the age of 18 years). We measured the drug concentration of infliximab from the patient’s blood sample, but the drug concentration was as low as 2.2 μg/mL, indicating insufficient drug efficacy (32, 33). In pediatric CD patients, dose intensification can be performed by advancing the IFX dosing interval to 6 or 4 weeks to increase drug level, but in the case of pediatric UC patients, there is no method of dose intensification. Fortunately, the joint pain is not severe enough to interfere with daily activities, and we are waiting for the patient to become an adult to switch to another biological agent.

This study has some limitations. First, due to the retrospective nature of this study, data collection was based on electronic medical records, which may have led to an underestimation of EIMs and have resulted in observational bias. We believe that the number of patients who first developed EIMs symptoms prior to the UC diagnosis may be greater. Nevertheless, we have sorted and selected data by applying the most objective method possible.

## Conclusion

Pediatric UC with EIMs had higher disease severity and often exhibited UGI tract involvement. In addition, even after the EIMs had been treated, new EIMs emerged repeatedly. Cumulative drug demand (steroids, immunosuppressants, and biological agents) for the treatment increased steadily over time, and frequent relapses occurred despite the use of many therapeutic drugs. Therefore, clinicians treating pediatric UC should consider performing EGD at the time of diagnosis and determine whether the patients display upper gastrointestinal symptoms. Since EIMs may precede intestinal symptoms, IBD should be suspected if there is a disease with unclear autoimmune features. In addition, patients who had had EIMs may develop new EIMs even after the treatment, so careful observation is required even at the time of follow-up. Since the demand for of therapeutic drugs is high for patients with EIMs, it is recommended to start high level of treatment from the beginning. Lastly, it is important to know about various treatment methods other than steroids due to the recent advances in the methodology of biological agents concentration measurement and the development of various biological agents. A prospective large-scale study of pediatric UC with EIMs will be needed in the future.

## Data Availability Statement

The datasets used and analyzed during the current study are available from the corresponding author on reasonable request.

## Ethics Statement

The studies involving human participants were reviewed and approved by the Institutional Review Board of Samsung Medical Center (IRB File No. SMC 2021-12-062). Written informed consent to participate in this study was provided by the participants’ legal guardian/next of kin.

## Author Contributions

MK and YC: conception or design. YK and EK: acquisition, analysis, or interpretation of data. YK and MK: drafting the work or revising and final approval of the manuscript. All authors read and approved the manuscript.

## Conflict of Interest

The authors declare that the research was conducted in the absence of any commercial or financial relationships that could be construed as a potential conflict of interest.

## Publisher’s Note

All claims expressed in this article are solely those of the authors and do not necessarily represent those of their affiliated organizations, or those of the publisher, the editors and the reviewers. Any product that may be evaluated in this article, or claim that may be made by its manufacturer, is not guaranteed or endorsed by the publisher.

## Abbreviations

UC: Ulcerative colitis
PUCAI: Pediatric ulcerative colitis activity index
IBD: Inflammatory bowel disease
EIM: Extraintestinal manifestation
CD: Crohn’s disease
ITP: Immunothrombocytopenia
UGI: Upper gastrointestinal
MES: Mayo endoscopic subscore.
